# Coping with complexity: working beyond the guidelines for patients with multimorbidities

**DOI:** 10.15256/joc.2015.5.49

**Published:** 2015-03-04

**Authors:** Julian Treadwell

**Affiliations:** ^1^Hindon Surgery, Wiltshire, UK; ^2^Royal College of General Practitioners’ Standing Group on Overdiagnosis, UK

**Keywords:** Multimorbidity, comorbidity, primary care, general practice, guidelines, over-treatment

Primary care physicians believe they are delivering evidence-based care, understanding that adherence to evidence-based clinical guidelines results in tangible benefits in the populations for which they were developed. Unfortunately, most clinical guidelines are based on trial populations which are very different to primary care populations [1], and do not reflect the reality of multimorbidity in general practice [2–6]. Since patients with multimorbidity account for around eight in every 10 primary care consultations [7], it is unsurprising that many primary care physicians find managing these patients challenging. Additionally, current clinical guidelines do not provide guidance on how best to prioritize recommendations for individuals with multimorbidity, and may therefore result in over-treatment and polypharmacy, and a risk of overlooking patient preferences [2, 8].

To illustrate the point, allow me to present Mary, an 82-year-old, socially active woman living alone. Mary has been taking alendronate and calcium/vitamin C following a Colles fracture 5 years ago. She has difficulty walking as a result of osteoarthritis (for which she takes paracetamol and naproxen) and chronic obstructive pulmonary disease (for which she uses salbutamol for short-term symptom relief plus a salmeterol/fluticasone inhaler to prevent exacerbations). Mary is visiting her GP today to discuss her recently diagnosed stage 2 hypertension (ambulatory blood pressure 162/92 mmHg), her fasting total:HDL (high-density lipoprotein cholesterol) of 5.3, and her newly diagnosed chronic kidney disease (CKD) stage 3aA2.

With strict adherence to all the current evidence-based guidelines for her conditions, Mary would be leaving her appointment today with a prescription containing all of the following:

- Paracetamol 1 g four-times per day, as needed
- Naproxen 250 mg twice-daily, as needed
- Calcichew D3 Forte two tablets per day
- Alendronate 70 mg once-weekly
- Salbutamol and/or ipratropium bromide inhalers, as needed
- Salmeterol/Fluticasone 50/500 mg inhaler, one puff twice-daily
- Atorvastatin 20 mg, once daily
- A calcium channel blocker, once daily
- An angiotensin-converting enzyme (ACE) inhibitor, possibly at the maximum dose
- Possibly a thiazide to achieve CKD blood pressure targets.

Patients like Mary are now the norm – not the exception – in primary care [5].

The Quality Outcomes Framework for general practice in the UK has resulted in more consistent use of evidence-based interventions, but has also led to far greater use of pharmacological therapies [9]. This has been compounded by a tendency towards over-diagnosis of premorbid disorders, with many patients receiving aggressive interventions for conditions that would never have harmed them [10, 11]. Polypharmacy is a risky business associated with causing or exacerbating other conditions, adverse drug reactions and interactions, under-dosing of recommended drugs, medication errors, and poor adherence to treatment [12].

In Mary’s circumstances and for many other patients, we need to adopt a more rational, patient-centered approach to care, and consider deviating from the guidelines when it is safe and justifiable to do so. Most guidelines contain a statement encouraging clinicians to exercise “clinical judgement”; to do this we need to consider what the individual patient really wants, prioritize those conditions most likely to do harm, and initiate only those treatments most likely to confer substantial benefits. We must make the best decisions we can in partnership with our patients.

Let us imagine that Mary expresses concern about the number of medications that she is being offered. She will do what her doctor advises, but would rather only take what is really necessary and wants to minimize her risk of side effects. Her treatment priorities are to reduce her risk of having a disabling stroke (a great fear) but she takes a philosophical approach about mortality and is not especially interested in taking drugs to increase longevity at her age; “You’ve got to die of something”. What her doctor needs to know now to help her make a treatment decision is: exactly how much good these treatments are likely to do her, how much harm they might cause, exactly how much risk reduction are they conferring, and a reduction in risk of what, exactly? An exploration of the evidence behind her guideline recommended treatment might lead her and her doctor to make some more prudent choices about her medication regime:

- Treatment of stage 2 hypertension has been shown to reduce the risk of stroke from 5.2% to 3.3% over approximately 4 years [13], and a similar degree of benefit was observed in the Hypertension in the Very Elderly (HYVET) trial [14] with a target blood pressure of <150/90 mmHg. A calcium channel blocker is the first class of drug recommended in current NICE hypertension guidance [15]. It would seem likely that Mary would opt for this treatment.
- An ACE inhibitor is recommended in current NICE guidance for CKD in the presence of moderate albuminuria and hypertension. Examination of the evidence for this recommendation reveals that this intervention is based on trials of patients with diabetes or severe, progressive CKD, and has been shown to reduce biochemical deterioration of CKD and the chance of end-stage CKD [16]. This is not a comparable population, nor a treatment goal that is likely to be relevant for Mary. Treatment with an ACE inhibitor exposes her to risks of hyperkalemia, hypotension, falls, and acute kidney injury.
- Why should she remain on her Salmeterol/­Fluticasone inhaler? In chronic obstructive pulmonary disease with an FEV1 (forced expiratory volume in 1 s) <50% of predicted, this is recommended to reduce her risk of exacerbations and hospital admissions [17]. However, the absolute gain for this is very small. A Cochrane review [18] reported a 2.1% absolute risk reduction in the composite endpoint of exacerbations and hospital admissions, but also a 1.4% increase in absolute risk of pneumonia, conferring a ‘net benefit’ of merely 0.7% absolute risk reduction. Would Mary think this a worthwhile exercise?
- Bisphosphonates have been shown to reduce the risk of osteoporotic fractures in a secondary prevention population [19], although the trial populations were younger than Mary and it is hard to extrapolate her likely gain from these drugs given the multifactorial nature of fracture risk. However, they are likely to be a valuable intervention for her. Nevertheless, given that she has already been taking them for 5 years, it may be reasonable to stop these treatments now as extension trials of bisphosphonates have not shown any longer term reduction in fracture risk [20]. Additionally, these drugs are associated with significant side effects, including gastrointestinal bleeding [21].
- There is little evidence about the benefit for her in continuing oral calcium and vitamin D supplements without bisphosphonates. A trial in a comparable population showed no benefit [22].
- The role of statins in the very elderly in a primary prevention context remains controversial. NICE guidance [23] recommends offering treatment but acknowledges the paucity of evidence in this age group. The PROSPER trial [24] in those aged over 70 years showed no benefit for stroke reduction or total mortality (though some reduction in fatal and non-fatal myocardial infarction was observed) with pravastatin 40 mg versus placebo.
- With regard to her analgesia, paracetamol and naproxen are both recommended in current NICE guidance [25], but Mary and her doctor would wish to know the size of the potential risks of non-steroidal anti-inflammatory drugs, which include GI bleeds, acute kidney injury, and cardiovascular events.

Given all this extra information, it may well be that Mary and her doctor choose to rationalize her long-term medication so that she might remain on just paracetamol, a calcium channel blocker and salbutamol inhaler as needed. In doing so, she may not lose much in terms of risk reduction, but gain a lot in reduced treatment burden, risk of acute kidney injury, GI bleed, hypotension, falls, and possibly hospital admission.

But how might we achieve this sort of personalized assessment in everyday practice? Gathering this amount of information is prohibitively time consuming and requires high levels of confidence on the part of the doctor interpreting it. Current clinical guidelines tend to be prescriptive and the evidence summaries behind them relatively inaccessible, do not consider the cumulative impact of treatment recommendations on people with multimorbidities, and do not state the potential risks and benefits of different treatments to the individual.

Primary care professionals are becoming increasingly frustrated by the prescriptive and additive nature of current guidelines based on research that excluded most of the types of patients with multimorbidities we see every day [8, 26–28]. Ideally, we would like to see clinical guidelines that are generalist-led, readily accessible and relevant to our work [2, 4]. We need a clinically meaningful evidence resource that draws on the excellent work presented in current guidelines, but would enable us to make prompt, patient-centred decisions based on an analysis of potential benefits and risk at the individual level. We would like to see data on absolute risk reductions with each intervention, numbers needed to treat in the context of a clear time frame, and numbers needed to harm clearly expressed, wherever possible. Breakdowns of composite endpoints would allow us to target treatment according to what the patient wants to achieve; for example, stroke versus non fatal myocardial infarction. We would like to have information about the applicability of study results to ‘real-world’ patients, with consideration given to the needs of different age groups, ethnicities, and patients with comorbidities/multimorbidities [29].

Primary care physicians need rapid access to evidence that matters – expressed in a way that is useful for patients – that can be found quickly during a consultation. A framework for a web-based resource is being explored by the Royal College of General Practitioners Standing Group on Overdiagnosis [30], and we hope to engage with others in the world of shared decision making to take this further. In the meantime, we should continue to be alert to the perils of polypharmacy in our patients with multimorbidity, aim to balance the risk of under-treatment against that of over-treatment, and to maximize use of the most important interventions that offer the greatest benefit with the smallest harm [9].

We have a long way to go before we are adequately equipped to provide evidence-based, patient-centered care for our patients with multimorbidity, but at least the issues of over-diagnosis and over-treatment are now on the agenda, and there is a palpable hunger for change in primary care in the UK.
